# Childhood brain tumours and use of mobile phones: comparison of a case–control study with incidence data

**DOI:** 10.1186/1476-069X-11-35

**Published:** 2012-05-20

**Authors:** Denis Aydin, Maria Feychting, Joachim Schüz, Martin Röösli

**Affiliations:** 1 Department of Epidemiology and Public Health, Swiss Tropical and Public Health Institute, Basel, Switzerland; 2 University of Basel, Basel, Switzerland; 3 Department of Epidemiology, Institute for Environmental Medicine, Karolinska Institutet, Stockholm, Sweden; 4 International Agency for Research on Cancer (IARC), Section of Environment and Radiation, Lyon, France

**Keywords:** Mobile phone, Brain tumour, Children, Adolescents, Incidence rates trends, CEFALO

## Abstract

The first case–control study on mobile phone use and brain tumour risk among children and adolescents (CEFALO study) has recently been published. In a commentary published in Environmental Health, Söderqvist and colleagues argued that CEFALO suggests an increased brain tumour risk in relation to wireless phone use. In this article, we respond and show why consistency checks of case–control study results with observed time trends of incidence rates are essential, given the well described limitations of case–control studies and the steep increase of mobile phone use among children and adolescents during the last decade. There is no plausible explanation of how a notably increased risk from use of wireless phones would correspond to the relatively stable incidence time trends for brain tumours among children and adolescents observed in the Nordic countries. Nevertheless, an increased risk restricted to heavy mobile phone use, to very early life exposure, or to rare subtypes of brain tumours may be compatible with stable incidence trends at this time and thus further monitoring of childhood brain tumour incidence rate time trends is warranted.

## Background

In a recent commentary [1], Söderqvist et al. discussed the findings of our international case–control study on mobile phone use and brain tumour risk in children and adolescents (CEFALO study) [2]. This is a response to their commentary.

## Result

**Figure 1:**
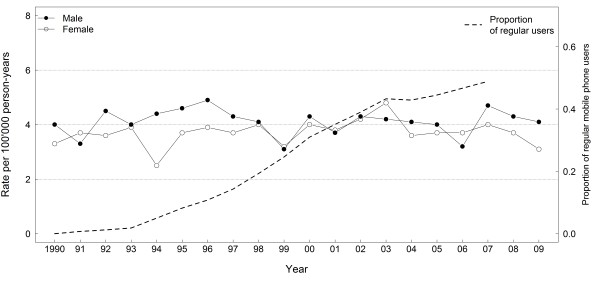
**Age-standardized incidence rates for brain and central nervous system tumours.** Rates are shown for the age group 5–19 years living in the Nordic Countries obtained from NORDCAN (http://www-dep.iarc.fr/nordcan/English/frame.asp], accessed January 9, 2012). Regular mobile phone use is defined as having had at least one call per week during a period of at least six months. We estimated the proportion of regular mobile phone users in this age group by combining data from the control subjects in CEFALO with subscriber data from Sweden.

Söderqvist et al. see “several indications of increased risk, despite low exposure, [and] short latency, …” [1] Nevertheless, they do not provide any explanation of how such an increased risk from use of wireless phones would correspond to relatively stable incidence time trends for brain tumours among children and adolescents in the Nordic countries over the last 20 years [Figure 1, and similarly stable incidence time trends in other countries [3-5]. As shown in Figure 1 the proportion of regular mobile phone users among children and adolescents has steeply increased over the last ten years. These data are even more incompatible if one considers their arguments that the risk estimates observed in CEFALO are underestimations of the true risk because of exposure misclassification due to cordless phone use and because of more widespread use of wireless phones among adolescents today compared to the time period when CEFALO was carried out (2004–2008). Regarding the inconsistency between results from analytical studies such as CEFALO and incidence time trends, the authors repeatedly imply that central nervous system tumours are underreported to the Swedish Cancer registry [1,6]. However, each time they fail to mention that the observed underreporting was mainly confined to patients 70 years or older [7], which has little relevance for incidence trends in children and adolescents, or other age-groups with high prevalence of mobile phone users.

As argued above, assuming a short latency of a few years, an increased brain tumour risk should be detectable in the incidence data that are already available today because of the steep increase in wireless phone use among adolescents during the last two decades (unless the risk is restricted to a very small subgroup of the population, e.g. very heavy mobile phone users). For this reason we restricted our analysis of cordless phone use to the first three years of use. Because most children and adolescents in CEFALO had used cordless phones earlier in life than mobile phones, we could address the effects of microwave radiation with longer latency time periods or with exposure at a young age. It is striking, however, that it was difficult for many participating families to recall the amount of cordless phone use early in life and some did not feel comfortable about answering questions about amount, duration, or years since first use. The seemingly inconsistent numbers in the tables are actually the consequences of missing answers to some of the questions [table six in the original paper [2]] or due to categories which were not mutually exclusive as explained in the footnotes in the original article [table four and five in the original paper].

## Discussion and Conclusion

The original conclusions in the abstract and the last paragraph of our paper [2] were that “the absence of an exposure–response relationship either in terms of the amount of mobile phone use or by localization of the brain tumour argues against a causal association.” And “we cannot, however, rule out the possibility that mobile phones confer a small increase in risk and therefore emphasize the importance of future studies with objective exposure assessment or the use of prospectively collected exposure data.” Meanwhile, all available data from epidemiological studies should be evaluated and discussed in a balanced way, taking into account the strengths and limitations of each respective study design. Because of the well-described limitations of case–control studies with retrospectively assessed self-reported wireless phone use, it is imperative to check the consistency of the observed relative risk estimates with observed time trends of incidence rates to avoid drawing wrong conclusions [8,9].

## Competing interests

The authors declare that they have no competing interests.

## Authors’ contributions

DA and MR drafted the manuscript. MF and JS commented and improved the manuscript. All authors read and approved the final manuscript.

## CEFALO study team

Tore Tynes, Tina Veje Andersen, Lisbeth Samsø Schmidt, Aslak Harbo Poulsen, Christoffer Johansen, Michaela Prochazka, Birgitta Lannering, Lars Klæboe, Tone Eggen, Daniela Jenni, Michael Grotzer, Nicolas Von der Weid, Claudia E. Kuehni.

## References

[B1] Söderqvist F Carlberg M Hansson Mild K Hardell L Childhood brain tumour risk and its association with wireless phones: a commentary Environ Health 2011 10 106 10.1186/1476-069X-10-106 22182218 PMC3278351

[B2] Aydin D Feychting M Schuz J Tynes T Andersen TV Schmidt LS Poulsen AH Johansen C Prochazka M Lannering B Mobile phone use and brain tumors in children and adolescents: a multicenter case–control study J Natl Cancer Inst 2011 103 1264 1276 10.1093/jnci/djr244 21795665

[B3] Boice JD Tarone RE Cell phones, cancer, and children J Natl Cancer Inst 2011 103 1211 1213 10.1093/jnci/djr285 21795667

[B4] de Vocht F Burstyn I Cherrie JW Time trends (1998–2007) in brain cancer incidence rates in relation to mobile phone use in England Bioelectromagnetics 2011 10.1002/bem.20648 21280060

[B5] Inskip PD Hoover RN Devesa SS Brain cancer incidence trends in relation to cellular telephone use in the United States Neuro Oncol 2010 12 1147 1151 10.1093/neuonc/noq077 20639214 PMC3098028

[B6] Hardell L Carlberg M Soderqvist F Mild KH Re: time trends in brain tumor incidence rates in Denmark, Finland, Norway, and Sweden, 1974–2003 J Natl Cancer Inst 2010 102 740 741 author reply 742–743 10.1093/jnci/djq122 20403845

[B7] Barlow L Westergren K Holmberg L Talback M The completeness of the Swedish Cancer Register: a sample survey for year 1998 Acta Oncol 2009 48 27 33 10.1080/02841860802247664 18767000

[B8] Deltour I Auvinen A Feychting M Johansen C Klaeboe L Sankila R Schüz J Mobile phone use and incidence of glioma in the nordic countries 1979–2008 Epidemiology 2012 23 epub ahead of print 10.1097/EDE.0b013e3182448295 22249239

[B9] Little MP Rajaraman P Curtis RE Devesa SS Inskip PD Check DP Linet MS Mobile phone use and glioma risk: comparison of epidemiological study results with incidence trends in the United States BMJ 2012 344 e1147 10.1136/bmj.e1147 22403263 PMC3297541

